# Epidemiology, causes, clinical manifestation and diagnosis, prevention and control of coronavirus disease (COVID-19) during the early outbreak period: a scoping review

**DOI:** 10.1186/s40249-020-00646-x

**Published:** 2020-03-17

**Authors:** Sasmita Poudel Adhikari, Sha Meng, Yu-Ju Wu, Yu-Ping Mao, Rui-Xue Ye, Qing-Zhi Wang, Chang Sun, Sean Sylvia, Scott Rozelle, Hein Raat, Huan Zhou

**Affiliations:** 1 West China School of Public Health and West China Fourth Hospital, Sichuan University, Chengdu, China; 2 Department of Communication Studies, California State University, Long Beach, CA 90802 USA; 3 Health Policy and Management, University of North Carolina at Chapel Hill, Chapel Hill, NC USA; 4 Freeman Spogli Institute for International Studies, Stanford University, Stanford, CA USA; 5 Department of Public Health, Erasmus MC—University Medical Center Rotterdam, 3000 CA Rotterdam, The Netherlands

**Keywords:** COVID-19, Epidemiology, Causes, Prevention and control, Review

## Abstract

**Background:**

The coronavirus disease (COVID-19) has been identified as the cause of an outbreak of respiratory illness in Wuhan, Hubei Province, China beginning in December 2019. As of 31 January 2020, this epidemic had spread to 19 countries with 11 791 confirmed cases, including 213 deaths. The World Health Organization has declared it a Public Health Emergency of International Concern.

**Methods:**

A scoping review was conducted following the methodological framework suggested by Arksey and O’Malley. In this scoping review, 65 research articles published before 31 January 2020 were analyzed and discussed to better understand the epidemiology, causes, clinical diagnosis, prevention and control of this virus. The research domains, dates of publication, journal language, authors’ affiliations, and methodological characteristics were included in the analysis. All the findings and statements in this review regarding the outbreak are based on published information as listed in the references.

**Results:**

Most of the publications were written using the English language (89.2%). The largest proportion of published articles were related to causes (38.5%) and a majority (67.7%) were published by Chinese scholars. Research articles initially focused on causes, but over time there was an increase of the articles related to prevention and control. Studies thus far have shown that the virus’ origination is in connection to a seafood market in Wuhan, but specific animal associations have not been confirmed. Reported symptoms include fever, cough, fatigue, pneumonia, headache, diarrhea, hemoptysis, and dyspnea. Preventive measures such as masks, hand hygiene practices, avoidance of public contact, case detection, contact tracing, and quarantines have been discussed as ways to reduce transmission. To date, no specific antiviral treatment has proven effective; hence, infected people primarily rely on symptomatic treatment and supportive care.

**Conclusions:**

There has been a rapid surge in research in response to the outbreak of COVID-19. During this early period, published research primarily explored the epidemiology, causes, clinical manifestation and diagnosis, as well as prevention and control of the novel coronavirus. Although these studies are relevant to control the current public emergency, more high-quality research is needed to provide valid and reliable ways to manage this kind of public health emergency in both the short- and long-term.

## Background

The coronavirus belongs to a family of viruses that may cause various symptoms such as pneumonia, fever, breathing difficulty, and lung infection [1]. These viruses are common in animals worldwide, but very few cases have been known to affect humans. The World Health Organization (WHO) used the term 2019 novel coronavirus to refer to a coronavirus that affected the lower respiratory tract of patients with pneumonia in Wuhan, China on 29 December 2019 [2–4]. The WHO announced that the official name of the 2019 novel coronavirus is coronavirus disease (COVID-19) [4]. And the current reference name for the virus is severe acute respiratory syndrome coronavirus 2 (SARS-CoV-2). It was reported that a cluster of patients with pneumonia of unknown cause was linked to a local Huanan South China Seafood Market in Wuhan, Hubei Province, China in December 2019 [5].

In response to the outbreak, the Chinese Center for Disease Control and Prevention (China CDC) dispatched a rapid response team to accompany health authorities of Hubei province and Wuhan city to conduct epidemiological and etiological investigations. The WHO confirmed that the outbreak of the coronavirus epidemic was associated with the Huanan South China Seafood Marketplace, but no specific animal association was identified [6]. Scientists immediately started to research the source of the new coronavirus, and the first genome of COVID-19 was published by the research team led by Prof. Yong-Zhen Zhang, on 10 January 2020 [7]. Within 1 month, this virus spread quickly throughout China during the Chinese New Year – a period when there is a high level of human mobility among Chinese people. Although it is still too early to predict susceptible populations, early patterns have shown a trend similar to Severe Acute Respiratory Syndrome (SARS) and Middle East respiratory syndrome (MERS) coronaviruses. Susceptibility seems to be associated with age, biological sex, and other health conditions [8]. COVID-19 has now been declared as a Public Health Emergency of International Concern by the WHO [9].

Given the spread of the new coronavirus and its impacts on human health, the research community has responded rapidly to the new virus and many preliminary research articles have already been published about this epidemic (Additional file 1). We conducted a scoping review to summarize and critically analyze all the published scientific articles regarding the new coronavirus in January 2020. This review aims to provide the evidence of early findings on the epidemiology, causes, clinical diagnosis, as well as prevention and control of COVID-19 in relation to time, location, and source of publication. This review can provide meaningful information for future research related to this topic and may support government decision-making on strategies to handle this public health emergency at the community, national, and international levels.

## Methods

### Study design

A scoping review was conducted following the methodological framework suggested by Arksey and O’Malley [10]. The following five steps were followed to conduct this scoping review: a) identifying a clear research objective and search strategies, b) identifying relevant research articles, c) selection of research articles, d) extraction and charting of data, and e) summarizing, discussing, analyzing, and reporting the results.

### Literature search strategies

Literature for this review was identified by searching the following online databases: bioRxiv, medRxiv, ChemRxiv, Google scholar, PubMed, as well as CNKI and WanFang Data (the two primary databases for biomedical research in mainland China). These online databases contain archives of most English and Chinese biomedical journals. In addition, some white papers published online by the National Health Commission of the People’s Republic of China, Chinese Center for Disease Prevention and Control, and the WHO were included in the analysis. We searched scientific publications from 1 January to 31 January 2020. The search terms were ‘nCoV’, ‘2019 novel coronavirus’, ‘2019-nCoV’, ‘novel coronavirus’, ‘Pneumonia’, ‘新型冠状病毒’ (Chinese), ‘新型肺炎’ (Chinese), and ‘新冠病毒’ (Chinese). We included all the relevant scientific publications written in English or Chinese in the review. Non-scientific commentary, reports, and news articles were excluded from the analysis.

### Identification and selection of relevant studies

**Fig. 1:**
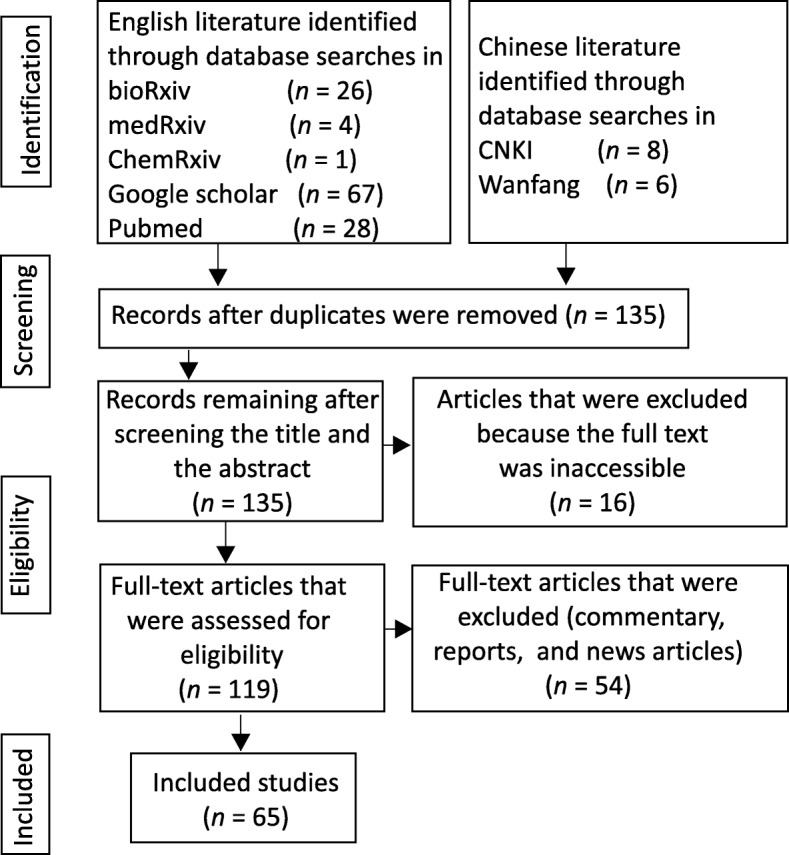
PRISMA flow diagram for the scoping review process

Two researchers (YW and SPA) independently searched through the literature. The two sets of literature were then compared. Disagreements on the inclusion or exclusion of literature were resolved through discussion or, if necessary, by including a third researcher (HZ) to make the final decision. Duplicate articles were eliminated. Eventually, 65 unique academic publications were included in this analysis (Additional file 1). Figure 1 presents a Preferred Reporting Items for Systematic Reviews and Meta-Analyses (PRISMA) flow diagram showing the process of searching and selecting the research articles [11].

### Data extraction from the included studies

After the articles were selected, data were extracted and recorded in an excel spreadsheet. The extracted data were date of publication, language of publication, title of article, name of journal, author’s country and affiliation, study design, targets of study, sample size, study setting, data collection instrument, research domain, and key findings.

### Summarizing the findings

Based on the main research objectives, articles were classified into one of the following four research domains: epidemiology, causes, clinical manifestation and diagnosis, or prevention and control. ‘Epidemiology’ includes studies on the epidemic distribution (when, where, who); ‘causes’ includes studies on virology, pathogenesis, and transmission patterns; ‘clinical manifestation and diagnosis’ includes studies on the clinical features (signs and symptoms) and diagnosis; and ‘prevention and control’ includes studies on prevention, control, and treatment measures.

Authors’ affiliations were used to categorize the type of research collaboration. A publication was considered as deriving from ‘inside China’ when all affiliations of the authors were from China; a publication was considered as deriving from ‘outside China’ when all affiliations were outside China; a publication was considered as deriving from ‘international collaboration’ when at least one affiliation was from China and at least one was from outside China. The research domains, publishing dates, journal language, authors’ affiliations, as well as methodological characteristics were analyzed respectively. All findings and statements regarding the outbreak in this review are based on published information as listed in the references.

## Results and discussion

### Characteristics of published studies

Among the 65 research articles included in the analysis, more than 31 are on preprint servers, and 34 are published in peer-reviewed journals, including *The Lancet* and *The New England Journal of Medicine*. Most of the publications (*n* = 58, 89.2%) are in English and few (*n* = 7, 10.8%) are in Chinese. Around 75.9% of English language publications focus on epidemiology and causes, while 85.7% of Chinese papers focus on prevention and control (Table 1).

**Table 1:** Breakdown of research domains by language in January 2020

Research domains	English literature		Chinese literature		Total	
	*n*	%	*n*	%	*n*	%
Epidemiology	19	32.8	0	0.0	19	29.2
Causes	25	43.1	0	0.0	25	38.5
Clinical manifestation and diagnosis	8	13.8	1	14.3	9	13.8
Prevention and control	6	10.3	6	85.7	12	18.5
Total	58	89.2	7	10.8	65	100

**Fig. 2:**
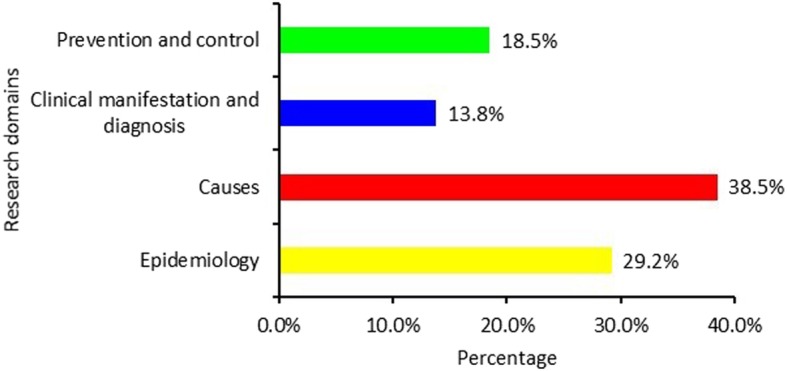
Research domains of published research articles

**Fig. 3:**
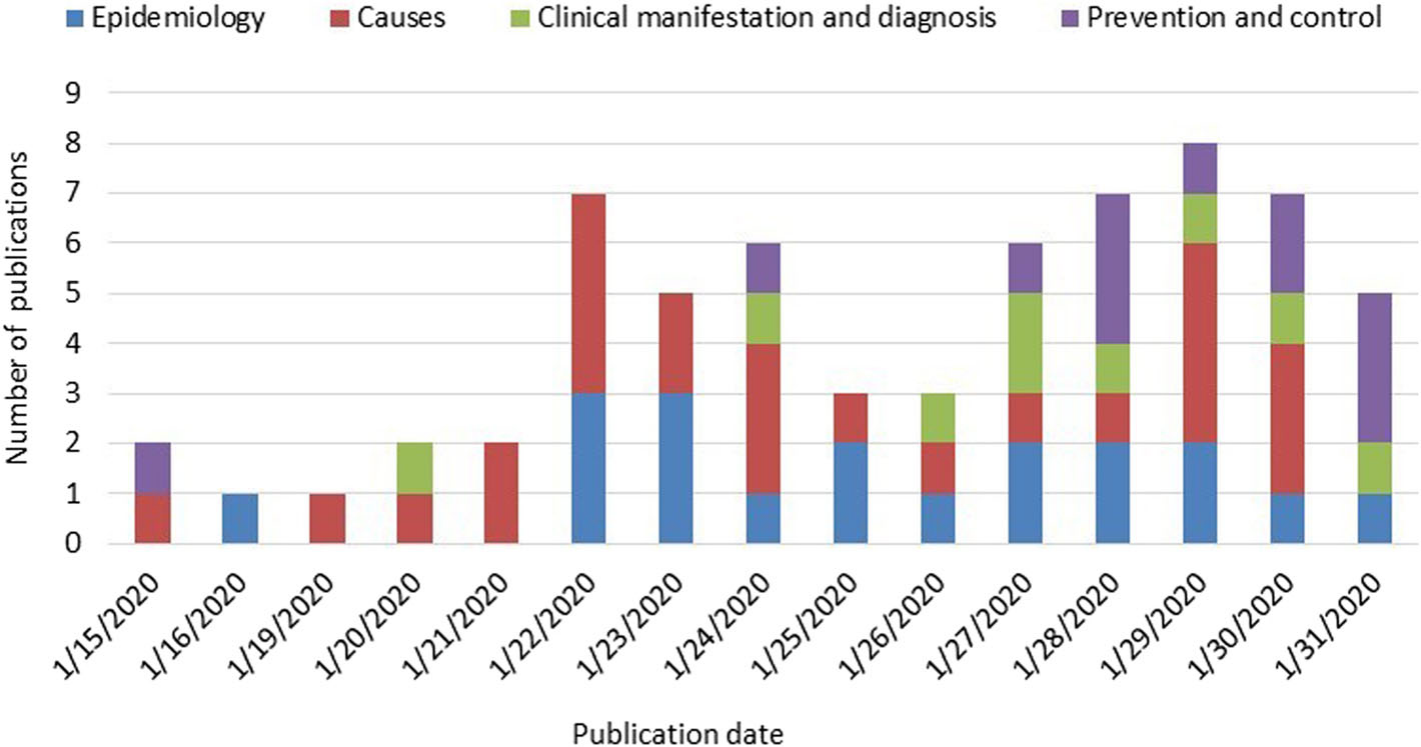
Publication dates of research articles

As shown in Fig. 2, academic publications are distributed across the following research domains: epidemiology, causes, clinical manifestation and diagnosis, and prevention and control. The largest portion of the papers (*n* = 25, 38.5%) are related to causes, followed by papers on epidemiology (*n* = 19, 29.2%), while 18.5% examined prevention and control and 13.8% reported clinical manifestations and diagnosis. Initially, there were more research articles focused on causes of the outbreak, yet studies on prevention and control gradually increased over time (Fig. 3). The majority of the articles (*n* = 44, 67.7%) were published by Chinese scholars whereas 29.2% (*n* = 19) articles were from scholars outside of China. A small number (*n* = 2, 3.1%) were based on international collaborative research by scholars from different countries (Table 2).

**Table 2:** Breakdown of research domains by collaboration type in January 2020

Research domains	Inside China		Outside China		International collaboration		Total	
	*n*	%	*n*	%	*n*	%	*n*	%
Epidemiology	8	18.2	9	47.4	2	100	19	29.2
Causes	17	38.6	8	42.1	0	0.0	25	38.5
Clinical manifestation and diagnosis	8	18.2	1	5.3	0	0.0	9	13.8
Prevention and control	11	25.0	1	5.3	0	0.0	12	18.5
Total	44	67.7	19	29.2	2	3.1	65	100

We also analyzed the methodological characteristics of the publications in the final sample. The majority of the articles are based on mathematical modeling (44.6%) and cross-sectional study designs (18.5%). Around half of the studies include targeted populations (patients, general populations, and healthcare workers) (49.2%) in their analysis, followed by studies that attempt to identify the species of the virus (virus structure, gene sequence) (36.9%). Among the 32 articles with population as the target of study, 34.4% had a sample size of less than 10, 31.3% were conducted in a hospital setting, and 50% used secondary data. Quality control activities for data collection were mentioned in 56.3% of the population studies (Table 3).

**Table 3:** Methodological characteristics of COVID-19 research articles in January 2020

Characteristics	Categories	*n*	%
Study design (*n* = 65)	Cross-sectional studies	12	18.5
	Mathematical modeling	29	44.6
	Molecular studies	10	15.4
	Diagnostic studies	2	3.1
	Review	3	4.6
	Theoretical study	9	13.9
Targets of study (*n* = 65)	Population	32	49.2
	Patients	22	33.9
	General population	6	9.2
	Healthcare workers	4	6.2
	Species (virus structure, gene sequence)	24	36.9
	Other	9	13.9
Study related to population (*n* = 32)			
Sample size	1–10	11	34.4
	11–50	5	15.6
	51–100	3	9.4
	> 100	3	9.4
	Not specified	10	31.3
Study setting	Laboratory	2	6.3
	Hospital	10	31.3
	Community	0	0.0
	Hospital and community	0	0.0
	Not specified	20	62.5
	Questionnaire	7	10.8
Data-collection instrument^a^	Biological specimen collection	1	3.1
	Physical examinations	0	0.0
	Environmental sample	0	0.0
	Hospital, medical, or exposure records	14	43.7
	Secondary data	16	50.0
Quality-control activities for data collection	Indicated in the article	14	56.3
	Not specified	18	43.8

^a^ Percentages in this section do not add up to 100% because multiple answers were possible

### Research domains

#### Epidemiology

On 29 December 2019, the first four cases of an acute respiratory syndrome of unknown etiology were reported in Wuhan City, Hubei Province, China among people linked to a local seafood market (“wet market”) [2]. Research is underway to understand more about transmissibility, severity, and other features associated with COVID-19 [3]. It appears that most of the early cases had some sort of contact history with the original seafood market [2, 12–14]. Soon, a secondary source of infection was found to be human-to-human transmission via close contact. There was an increase of infected people with no history of exposure to wildlife or visiting Wuhan, and multiple cases of infection were detected among medical professionals [2, 14–17]. It became clear that the COVID-19 infection occurs through exposure to the virus, and both the immunosuppressed and normal population appear susceptible. Some studies have reported an age distribution of adult patients between 25 and 89 years old. Most adult patients were between 35 and 55 years old [14], and there were fewer identified cases among children and infants [14, 18]. A study on early transmission dynamics of the virus reported the median age of patients to be 59 years, ranging from 15 to 89 years, with the majority (59%) being male [2]. It was suggested that the population most at risk may be people with poor immune function such as older people and those with renal and hepatic dysfunction [2].

The COVID-19 has been found to have higher levels of transmissibility and pandemic risk than the SARS-CoV, as the effective reproductive number (R) of COVID-19 (2.9) is estimated to be higher than the reported effective reproduction number (R) of SARS (1.77) at this early stage [15]. Different studies of COVID-19 have estimated the basic reproduction (R_0_) range to be from 2.6 to 4.71 (Table 4). The average incubation duration of COVID-19 was estimated to be 4.8 ± 2.6, ranging from 2 to 11 days [15] and 5.2 days (95% confidence interval, 4.1 to 7) [2]. The latest guidelines from Chinese health authorities stated an average incubation duration of 7 days, ranging from 2 to 14 days [23]. Table 4 summarizes the findings on important indicators from these epidemiological studies.

**Table 4:** Main epidemiological indicators of COVID-19 research articles in January 2020

Indicators	Description
Age of patients	• Cases range between 25 and 89 years, with most patients aged between 35 and 55 years and fewer cases among children and infants [14]
	• Median age of patients is 59 years, ranging from 51 to 89 [2]
	• Average age of patients was 55.5 years; age distribution: ≤ 39: 10%; 40–49: 22%, 50–59: 30%; 60–69: 22%, ≥ 70: 15% [19]
	• Cases range from 2 to 72 years [20]
Sex of patients	• More cases were males [20]
	• 59% males [2]
	• 68% males [19]
Age of the deaths	• Median age of death was 75 (with a range between 48 and 89 years) [21]
Exposure history	• Huanan Seafood Market in Wuhan [19, 22]
	• Wuhan residents or people who visited Wuhan [20]
Incubation time	• 4.8 ± 2.6 days (2–11 days) [15]
	• 5.2 days (4.1–7 days) [2]
	• Average of 7 days (2–14 days) [23]
	• Average of 10 days [22]
	• 5–6 days [24]
	• Average of 6.4 days (5.6–7.7 days) [20]
Basic Reproduction (R_0_)	• 2.6 (uncertainty range: 1.5–3.5) [25]
	• 3.8 (95% *CI*: 3.6–4.0) [26]
	• 2.2 (1.4–3.8) [27]
	• 4.71 (4.50–4.92) [24]
	• 2.68 (95% *CI*: 2.47–2.86) [28]
Susceptible populations	• Elderly people [21]
	• People with poor immune function [2]
	• People with chronic co-morbidities [2, 15, 19, 21]
	• People with long-term use of immunosuppressive agents [19]
	• Surgery history before admission [21]
Mortality rate	• 3% (between 29 December 2019 to 23 January, 2020) [15]
	• 2.3% (as of 28 January 2020) [29]
	• 2.8% (as of 25 January, 2020) [21]
	• 2.9% (as of 25 January, 2020) [30]
	• 11% (as of 25 January, 2020) [19]
	• 3.1% (as of 24 January 2020) [31]

**Fig. 4:**
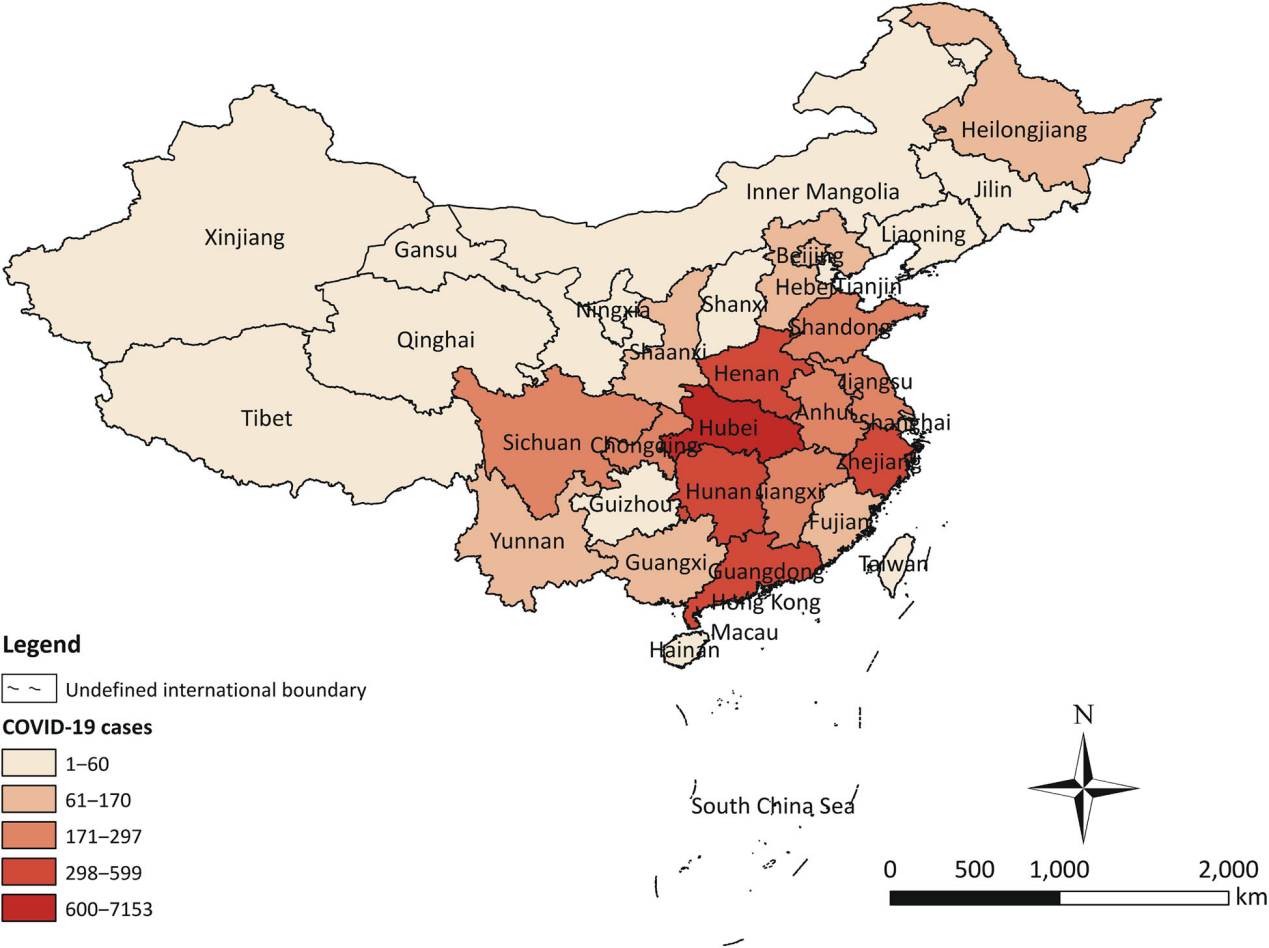
Distribution of COVID-19 cases in China

**Fig. 5:**
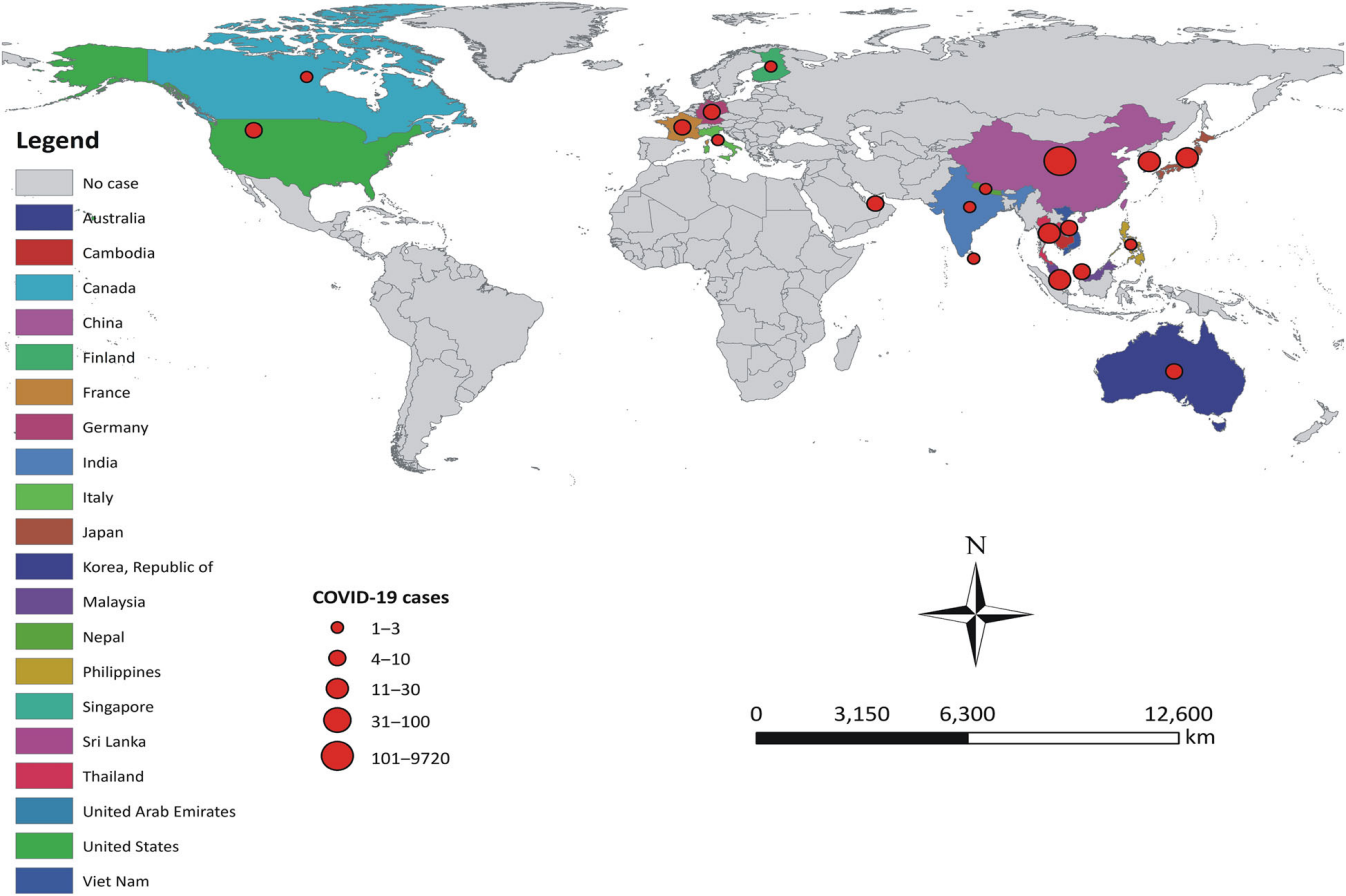
Global distribution of COVID-19 cases

In China, 11 791 cases were confirmed and 17 988 cases were suspected in 34 provinces as of 24:00, 31 January 2020 (Fig. 4) [32]. Studies indicated that the spread of COVID-19 was relatively quick and reported that it had spread to several other countries after its outbreak in China. On 31 January 2020, there were 213 deaths reported globally [33]. Confirmed cases were reported in the following 19 countries outside of China: Australia (9), Canada (3), Cambodia (1), France (6), Finland (1), Germany (5), India (1), Italy (2), Japan (14), Nepal (1), Malaysia (8), the Philippines (1), the Republic of Korea (11), Singapore (13), Sri Lanka (1), Thailand (14), the United States of America (6), United Arab Emirates (4) and Vietnam (5) (Fig. 5) [33].

#### Causes

##### Virology and pathogenesis

Coronaviruses are enveloped single-stranded RNA viruses that are zoonotic in nature and cause symptoms ranging from those similar to the common cold to more severe respiratory, enteric, hepatic, and neurological symptoms [5, 34]. Other than SARS-CoV-2, there are six known coronaviruses in humans: HCoV-229E, HCoV-OC43, SARS-CoV, HCoV-NL63, HCoV-HKU1, and MERS-CoV [2, 23, 35, 36]. Coronavirus has caused two large-scale pandemics in the last two decades: SARS [37] and MERS [12, 38].

To detect the infection source of COVID-19, China CDC researchers collected 585 environmental samples from the Huanan Seafood Market in Wuhan, Hubei Province, China on 1 January and 12 January 2020. They detected 33 samples containing SARS-CoV-2 and indicated that it originated from wild animals sold in the market [39]. Then, researchers used the lung fluid, blood, and throat swab samples of 15 patients to conduct laboratory tests. These laboratory tests found that the virus-specific nucleic acid sequences in the sample are different from those of known human coronavirus species. Laboratory results also indicated that SARS-CoV-2 is similar to some of the beta (β) coronaviruses genera identified in bats [12, 18, 40], which is situated in a group of SARS/SARS-like CoV [12].

To conduct next-generation sequencing from bronchoalveolar lavage fluid and cultured isolates, researchers enrolled nine inpatients in Wuhan with viral pneumonia and negative in common respiratory pathogens. The results of this next-generation sequencing indicated that SARS-CoV-2 was more distant from SARS-CoV (with about 79% sequence identity) and MERS-CoV (with about 50% sequence identity) than from two bat-derived SARS-like coronaviruses – bat-SL-CoVZC45 (with 87.9% sequence identity) and bat-SL-CoVZXC21 (with 87.2% sequence identity) [41]. Studies also reported that COVID-19 S-protein supported strong interaction with human ACE2 molecules despite the dissimilarity of its sequence with that of SARS-CoV [12, 42].

##### Transmission pattern

Many domestic and wild animals, including camels, cattle, cats, and bats, may serve as hosts for coronaviruses [23]. It is considered that, generally, animal coronaviruses do not spread among humans [3]. However, there are exceptions, such as SARS and MERS, which are mainly spread though close contact with infected people via respiratory droplets from cough or sneezing. With regard to COVID-19, early patients were reported to have some link to the Huanan Seafood Market in Wuhan, China, suggesting that these early infections were due to animal-to-person transmission. However, later cases were reported among medical staff and others with no history of exposure to that market or visiting Wuhan, which was taken as an indication of human-to-human transmission [2, 4, 15–17].

The latest guidelines from Chinese health authorities [23, 43] described three main transmission routes for the COVID-19: 1) droplets transmission, 2) contact transmission, and 3) aerosol transmission. Droplets transmission was reported to occur when respiratory droplets (as produced when an infected person coughs or sneezes) are ingested or inhaled by individuals nearby in close proximity; contact transmission may occur when a subject touches a surface or object contaminated with the virus and subsequently touch their mouth, nose, or eyes; and aerosol transmission may occur when respiratory droplets mix into the air, forming aerosols and may cause infection when inhaled high dose of aerosols into the lungs in a relatively closed environment [23, 43]. In addition to these three routes, one study also indicated the digestive system as a potential transmission route for COVID-19 infection. Since patients had abdominal discomfort and diarrhea symptoms, researchers analyzed four datasets with single-cell transcriptomes of digestive systems and found that ACE2 was highly expressed in absorptive enterocytes from ileum and colon [44].

#### Clinical manifestation and diagnosis

The complete clinical manifestation is not clear yet, as the reported symptoms range from mild to severe, with some cases even resulting in death [3]. The most commonly reported symptoms are fever, cough, myalgia or fatigue, pneumonia, and complicated dyspnea, whereas less common reported symptoms include headache, diarrhea, hemoptysis, runny nose, and phlegm-producing cough [3, 16]. Patients with mild symptoms were reported to recover after 1 week while severe cases were reported to experience progressive respiratory failure due to alveolar damage from the virus, which may lead to death [13]. Cases resulting in death were primarily middle-aged and elderly patients with pre-existing diseases (tumor surgery, cirrhosis, hypertension, coronary heart disease, diabetes, and Parkinson’s disease) [13]. Case definition guidelines mention the following symptoms: fever, decrease in lymphocytes and white blood cells, new pulmonary infiltrates on chest radiography, and no improvement in symptoms after 3 days of antibiotics treatment [2].

For patients with suspected infection, the following procedures have been suggested for diagnosis: performing real-time fluorescence (RT-PCR) to detect the positive nucleic acid of SARS-CoV-2 in sputum, throat swabs, and secretions of the lower respiratory tract samples [13, 14, 43].

#### Prevention and control

Prevention and control strategies and methods are reported at three levels: national level, case-related population level, and general population level. At the national level, the National Health Commission of the People’s Republic of China issued the “No.1 announcement” on 20 January 2020, which officially included the COVID-19 into the management of class B legal infectious diseases, and allowed for class A infectious disease preventive and control measures to be implemented [45]. Under this policy, medical institutes can adopt isolation treatment and observation protocols to prevent and control the spread of the COVID-19. On 22 January 2020, the National Health Commission published national guidelines for the prevention and control of COVID-19 for medical institutes to prevent nosocomial infection [46]. On 28 January 2020, the National Health Commission issued protocols for rapid prevention and control measures in order to effectively contain the spread of the epidemic through a “big isolation and big disinfection” policy during the Chinese Spring Festival [47]. National-level strategies have also been issued with targeted measures for rural areas (issued on 28 January 2020) and the elderly population (issued on 31 January 2020) [48, 49]. Several public health measures that may prevent or slow down the transmission of the COVID-19 were introduced; these include case isolation, identification and follow-up of contacts, environmental disinfection, and use of personal protective equipment [50].

To date, no specific antiviral treatment has been confirmed to be effective against COVID-19. Regarding patients infected with COVID-19, it has been recommended to apply appropriate symptomatic treatment and supportive care [3, 16]. There are six clinical trials registered in both the International Clinical Trials Registry platform and the Chinese Clinical Trial Registry to evaluate the efficacy or safety of targeted medicine in the treatment or prognosis of COVID-19 (Additional file 2) [51, 52]. Regarding infected patients with COVID-19, it has been recommended to apply appropriate symptomatic treatment and supportive care [3, 16]. Studies have also explored the prevention of nosocomial infection and psychological health issues associated with COVID-19. A series of measures have been suggested to reduce nosocomial infection, including knowledge training for prevention and control, isolation, disinfection, classified protections at different degrees in infection areas, and protection of confirmed cases [18, 50, 53]. Concerning psychological health, some suggested psychological intervention for confirmed cases, suspected cases, and medical staff [18, 54].

For the general population, at this moment there is no vaccine preventing COVID-19. The best prevention is to avoid being exposed to the virus [55]. Airborne precautions and other protective measures have been discussed and proposed for prevention. Infection preventive and control (IPC) measures that may reduce the risk of exposure include the following: use of face masks; covering coughs and sneezes with tissues that are then safely disposed of (or, if no tissues are available, use a flexed elbow to cover the cough or sneeze); regular hand washing with soap or disinfection with hand sanitizer containing at least 60% alcohol (if soap and water are not available); avoidance of contact with infected people and maintaining an appropriate distance as much as possible; and refraining from touching eyes, nose, and mouth with unwashed hands [3].

The WHO also issued detailed guidelines on the use of face masks in the community, during care at home, and in the health care settings of COVID-19 [56]. In this document, health care workers are recommended to use particulate respirators such as those certified N95 or FFP2 when performing aerosol-generating procedures and to use medical masks while providing any care to suspected or confirmed cases. According to this guideline, individuals with respiratory symptoms are advised to use medical masks both in health care and home care settings properly following the infection prevention guidelines. According to this guideline, an individual without respiratory symptoms is not required to wear a medical mask when in public. Proper use and disposal of masks is important to avoid any increase in risk of transmission [56].

In addition to articles published in research journals, the China CDC published a guideline to raise awareness of the prevention and control of COVID-19 among the general population. The key messages of the guideline include causes, how to choose and wear face masks, proper hand washing habits, preventive measures at different locations (e.g., at home, on public transportation, and in public space), disinfection methods, and medical observation at home [57]. In addition to scientific knowledge on ways to handle the COVID-19 outbreak, the guideline also suggests ways to eliminate panic among the general population [57].

### Strengths and limitations of the study

The review applied a systematic and rigorous search strategy to retrieve relevant articles according to the research objectives. This research summarizes scientific foundations, identifies literature gaps, and suggests some evidence for future research directions on COVID-19 which will provide information for research community, policymakers, and health professionals to adjust and/or come up with new research, policies, and practices. Our study only focuses on the articles published either in English or in Chinese during the early outbreak period. Although, it cannot reflect the entire body of research on COVID-19 worldwide, it will provide some evidences for future study and control.

## Conclusions

This study shows a holistic picture of the current research in response to the outbreak of COVID-19. During this early period, many studies have been published exploring the epidemiology, causes, clinical manifestation and diagnosis, and prevention and control of the novel coronavirus. Thus far, most studies have focused on the epidemiology and potential causes. However, studies exploring prevention and control measures have begun to gradually increase. Studies in this domain are urgently needed to minimize the impact of the outbreak. Government agencies have quickly incorporated recent scientific findings into public policies at the community, regional, and national levels to slow down and/or prevent the further spread of the COVID-19. We recommend that the scholarly community conduct further research to provide valid and reliable ways to manage this kind of public health emergency in both the short-term and long-term.

## Supplementary information

**Additional file 1.** Published research articles on COVID-19 in January 2020.

**Additional file 2.** Clinical trials registered as of 31 January 2020.

## Data Availability

The dataset supporting the conclusions of this article is included as an Additional file table.

## Abbreviations

ACE2: Angiotensin converting enzyme 2
CDC: Center for Disease Control
IPC: Infection prevention and control
MERS: Middle East Respiratory Syndrome
PRISMA: Preferred Reporting Items for Systematic Reviews and Meta-Analyses
SARS: Severe Acute Respiratory Syndrome
WHO: World Health Organization
