# Supplementary material for: Epidemiology, causes, clinical manifestation and diagnosis, prevention and control of coronavirus disease (COVID-19) during the early outbreak period: a scoping review
Source: Infect Dis Poverty. 2020 Mar 17;9:29. doi: 10.1186/s40249-020-00646-x (PMC7079521; doi:10.1186/s40249-020-00646-x)
Supplement: Supplementary file 1 — Additional file 1. Published research articles on COVID-19 in January 2020. [file 40249_2020_646_MOESM1_ESM.docx]

| **Additional file 1** Published research articles on COVID-19 in January 2020 | | | | | |
| --- | --- | --- | --- | --- | --- |
| No. | Date | Title | Journal name | Language | First author |
| 1 | 1/15/2020 | Pathogenicity and control of coronavirus | Journal of Microbes and infection | Chinese | Yumei Wen |
| 2 | 1/15/2020 | Recent advances in the detection of respiratory virus infection in humans | Journal of medical virology | English | Naru Zhang |
| 3 | 1/16/2020 | Outbreak of Pneumonia of Unknown Etiology in Wuhan China: the Mystery and the Miracle | Journal of medical virology | English | Hongzhou Lu |
| 4 | 1/20/2020 | Genomic and protein structure modelling analysis depicts the origin and infectivity of 2019-nCoV, a new coronavirus which caused a pneumonia outbreak in Wuhan, China | bioRxiv | English | Ning Dong |
| 5 | 1/20/2020 | Genomic characterization of the 2019 novel human-pathogenic coronavirus isolated from a patient with atypical pneumonia after visiting Wuhan | Emerging microbes & infections | English | Jasper Fuk-Woo Chan |
| 6 | 1/21/2020 | Host and infectivity prediction of Wuhan 2019 novel coronavirus using deep learning algorithm | bioRxiv | English | Qian Guo |
| 7 | 1/21/2020 | Evolution of the novel coronavirus from the ongoing Wuhan outbreak and modeling of its spike protein for risk of human transmission | SCIENCE CHINA Life Sciences | English | Xintian Xu, Ping Chen, Jingfang Wang |
| 8 | 1/22/2020 | Functional assessment of cell entry and receptor usage for lineage B β-coronaviruses, including 2019-nCoV | bioRxiv | English | Michael Letko |
| 9 | 1/22/2020 | Homologous recombination within the spike glycoprotein of the newly identified coronavirus may boost cross‐species transmission from snake to human | Journal of medical virology | English | Wei Ji |
| 10 | 1/22/2020 | Coronaviruses: genome structure, replication, and pathogenesis. | Journal of medical virology | English | Yu Chen |
| 11 | 1/22/2020 | Emerging coronaviruses: genome structure, replication, and pathogenesis | Journal of medical virology | English | Chen Y |
| 12 | 1/22/2020 | Estimating the potential total number of novel Coronavirus cases in Wuhan City China | Preprint published by the Imperial College London | English | Natsuko Imai |
| 13 | 1/22/2020 | Report 2: Estimating the potential total number of novel Coronavirus cases in Wuhan City, China | Preprint published bythe Imperial College London | English | Natsuko Imai |
| 14 | 1/22/2020 | Report 3: Transmissibility of 2019-nCoV | Imperial College and London | English | Natsuko Imai |
| 15 | 1/23/2020 | Detection of 2019 novel coronavirus (2019-nCoV) by real-time RT-PCR | Euro Surveill | English | Victor M Corman |
| 16 | 1/23/2020 | Discovery of a novel coronavirus associated with the recent pneumonia outbreak in humans and its potential bat origin | bioRxiv | English | Peng Zhou |
| 17 | 1/23/2020 | Novel coronavirus 2019-nCoV : early estimation of epidemiological parameters and epidemic prediction | medRxiv | English | Jonathan M. Read |
| 18 | 1/23/2020 | Pattern of early human-to-human transmission of Wuhan 2019-nCoV | bioRxiv | English | Julien Riou |
| 19 | 1/23/2020 | Modelling the epidemic trend of the 2019 novel coronavirus outbreak in China | bioRxiv | English | Mingwang Shen |
| 20 | 1/24/2020 | A Novel Coronavirus from Patients with Pneumonia in China, 2019 | The New England journal of medicine | English | Na Zhu |
| 21 | 1/24/2020 | Complete genome characterisation of a novel coronavirus associated with severe humanrespiratory disease in Wuhan, China | bioRxiv | English | Fan Wu |
| 22 | 1/24/2020 | The extent of transmission of novel coronavirus in Wuhan, China, 2020 | Journal of clinical medicine | English | Hiroshi Nishiura |
| 23 | 1/24/2020 | A familial cluster of pneumonia associated with the 2019 novel coronavirus indicating person-to-person transmission: a study of a family cluster | Lancet | English | Jasper Fuk-Woo Chan |
| 24 | 1/24/2020 | Clinical features of patients infected with 2019 novel coronavirus in Wuhan, China | Lancet (London, England) | English | Chaolin Huang |
| 25 | 1/24/2020 | 2019-20 Wuhan coronavirus outbreak:Intense surveillance is vital for preventing sustained transmission in newlocations | bioRxiv | English | R.N. Thompson |
| 26 | 1/25/2020 | Origin time and epidemic dynamics of the 2019 novel coronavirus | bioRxiv | English | Chi Zhang |
| 27 | 1/25/2020 | Coronavirus Infections and Immune Responses | Journal of Medical Virology | English | Geng Li |
| 28 | 1/25/2020 | Preliminary risk analysis of 2019 novel coronavirus spread within and beyond China Watts, Kamran Khan, Zhongjie | bioRxiv | English | Shengjie Lai |
| 29 | 1/26/2020 | Full-genome evolutionary analysis of the novel corona virus (2019-nCoV) rejects the hypothesis of emergence as a result of a recent recombination event | bioRxiv | English | D. Paraskevis |
| 30 | 1/26/2020 | Single-cell RNA expression profiling of ACE2, the putative receptor of Wuhan 2019-nCov | bioRxiv | English | Yu Zhao |
| 31 | 1/26/2020 | Transmission dynamics of 2019 novel coronavirus (2019-nCoV) | bioRxiv | English | Tao Liu |
| 32 | 1/27/2020 | Nelfinavir was predicted to be a potential inhibitor of 2019-nCov main protease by an integrative approach combining homology modelling, molecular docking and binding free energy calculation | bioRxiv | English | Zhijian Xu |
| 33 | 1/27/2020 | Use of the informational spectrum methodology for rapid biological analysis of the novel coronavirus 2019-nCoV: prediction of potential receptor, natural reservoir, tropism and therapeutic/vaccine target | F1000Research | English | Veljko Veljkovic |
| 34 | 1/27/2020 | The incubation period of 2019-nCoV infections among travellers from Wuhan, China | medRxiv | English | Jantien A. Backer |
| 35 | 1/27/2020 | Epidemiological and Clinical Characteristics of 99 Cases of 2019-Novel Coronavirus (2019-nCoV) Pneumonia in Wuhan, China | lancet | English | Nanshan Chen |
| 36 | 1/27/2020 | Coronavirus: A Mini-Review | International Journal of Current Research in Medical Science | English | Ashikujaman Syed |
| 37 | 1/27/2020 | Breaking down of the healthcare system: Mathematical modelling for controlling the novel coronavirus(2019-nCoV) outbreak in Wuhan, China | bioRxiv | English | Wai-Kit Ming |
| 38 | 1/28/2020 | Potent binding of 2019 novel coronavirus spike protein by a SARS coronavirus-specific human monoclon alantibody | bioRxiv | English | Xiaolong Tian |
| 39 | 1/28/2020 | Sofosbuvir Can Inhibit the Newly Emerged Coronavirus (2019-nCoV) in Wuhan, China | Lancet | English | Abdo A Elfiky |
| 40 | 1/28/2020 | Beware of asymptomatic transmission: Study on 2019-nCoV prevention and control measures based on extended SEIR model | bioRxiv | English | Peng Shao |
| 41 | 1/28/2020 | Therapeutic Drugs Targeting 2019-nCoV Main Protease by High-Throughput Screening | bioRxiv | English | Yan Li |
| 42 | 1/28/2020 | Drug treatment options for the 2019-new coronavirus (2019-nCoV) | Bioscience trends | English | Hongzhou Lu |
| 43 | 1/28/2020 | Epidemiological characteristics of novel coronavirus infection: A statistical analysis of publicly available case data | medRxiv | English | Natalie M. Linton |
| 44 | 1/28/2020 | Epidemiological identification of a novel infectious disease in real time: Analysis of the atypical pneumonia outbreak in Wuhan, China, 2019-20 | medRxiv | English | Sung-mok Jung |
| 45 | 1/29/2020 | Receptor recognition by novel coronavirus from Wuhan: An analysis based on decade-long structural studies of SARS | Journal of virology | English | Yushun Wan |
| 46 | 1/29/2020 | The 2019-new coronavirus epidemic: evidence for virus evolution | Journal of medical virology | English | Domenico Benvenuto |
| 47 | 1/29/2020 | Pneumonia of Unknown Etiology in Wuhan, China: Potential for International Spread Via Commercial Air Travel | Journal of Travel Medicine | English | Isaac I. Bogoch |
| 48 | 1/29/2020 | Potential inhibitors for 2019-nCoV coronavirus M protease from clinically approved medicines | bioRxiv | English | Xin Liu |
| 49 | 1/29/2020 | Clinical characteristics and syndrome differentiation of new coronavirus (2019-nCoV) pneumonia in traditional Chinese medicine | Journal of Traditional Chinese Medicine | Chinese | Yuguang Wang |
| 50 | 1/29/2020 | Epidemiological and clinical characteristics of 99 cases of 2019 novel coronavirus pneumonia in Wuhan, China: a descriptive study | The Lancet | English | Nanshan Chen |
| 51 | 1/29/2020 | Early Transmission Dynamics in Wuhan, China, of Novel Coronavirus-Infected Pneumonia | The New England journal of medicine | English | Qun Li |
| 52 | 1/29/2020 | Updated understanding of the outbreak of 2019 novel coronavirus (2019-nCoV) in Wuhan, China | Journal of medical virology | English | Weier Wang |
| 53 | 1/30/2020 | Evolution and variation of 2019-novel coronavirus | bioRxiv | English | Chenglong Xiong |
| 54 | 1/30/2020 | Uncanny similarity of unique inserts in the 2019-nCoV spike protein to HIV-1 gp120 and Gag | bioRxiv | English | Prashant Pradhan |
| 55 | 1/30/2020 | Potential of large 'first generation' human-to-human transmission of 2019-nCoV | Journal of medical virology | English | Xingguang LI |
| 56 | 1/30/2020 | Suggestions on management strategies of pregnant women infected with new coronavirus (2019 ncov) in Henan Province | Journal of Zhengzhou University(Medical Sciences) | Chinese | Xinyan Wang |
| 57 | 1/30/2020 | Identification of a novel coronavirus causing severe pneumonia in human: a descriptive study | Chinese Medical Journal | English | Li-Li Ren |
| 58 | 1/30/2020 | Preliminary estimation of the basic reproduction number of novel coronavirus (2019-nCoV) in China, from 2019 to 2020: A data-driven analysis in the early phase of the outbreak | bioRxiv | English | Shi Zhao |
| 59 | 1/30/2020 | Investigation on the psychological status of the first batch of clinical first-line support nurses to fight against pneumonia caused by novel coronavirus. | Chinese Nursing Reserch | Chinese | Mingchuan Xu |
| 60 | 1/31/2020 | Countermeasures for rapid spread of new coronavirus pneumonia in Wuhan | Chinese General Practicing Nursing | Chinese | Fen Ouyang |
| 61 | 1/31/2020 | The digestive system is a potential route of 2019-nCov infection: a bioinformatics analysis based on single-cell transcriptomes | bioRxiv | English | Hao Zhang |
| 62 | 1/31/2020 | Nowcasting and forecasting the potential domestic and international spread of the 2019-nCoV outbreak originating in Wuhan, China: a modelling study | The Lancet | English | Joseph T Wu, Kathy Leung |
| 63 | 1/31/2020 | Application effect of hazard vulnerability analysis in cope with 2019 novel coronavirus transmission in non⁃closed hematology ward | Nursing Research of China | Chinese | Chen Bin |
| 64 | 1/31/2020 | Prevalence, nosocomial infection and psychological prevention of novel coronavirus infection | Chinese General Practice Nursing | Chinese | Wang C |
| 65 | 2020/1/19 | A mathematical model for simulating the transmission of Wuhan novel Coronavirus | bioRxiv | English | Tianmu Chen |
