# Supplementary material for: Epidemiology, causes, clinical manifestation and diagnosis, prevention and control of coronavirus disease (COVID-19) during the early outbreak period: a scoping review
Source: Infect Dis Poverty. 2020 Mar 17;9:29. doi: 10.1186/s40249-020-00646-x (PMC7079521; doi:10.1186/s40249-020-00646-x)
Supplement: Supplementary file 2 — Additional file 2. Clinical trials registered as of 31 January 2020. [file 40249_2020_646_MOESM2_ESM.docx]

**Additional file 2** Clinical trials registered as of 31 January 2020

| No. | Public title | Applicant's institution | Registration date |
| --- | --- | --- | --- |
| 1 | A randomized, open-label, blank-controlled trial for the efficacy and safety of lopinavir-ritonavir and interferon-alpha 2b in hospitalization patients with novel coronavirus pneumonia (COVID-19) | Wuhan Jinyintan Hospital (Wuhan Infectious Diseases Hospital) | 2020/1/23 |
|  |  |  |  |
| 2 | A prospective comparative study for Xue-Bi-Jing injection in the treatment of novel coronavirus pneumonia (COVID-19) | The First Affiliated Hospital of Guangzhou Medical University | 2020/1/27 |
|  |  |  |  |
| 3 | Adjunctive Corticosteroid Therapy for Patients with Severe Novel Coronavirus Pneumonia (COVID-19): a Randomized Controlled Trial | Chongqing Public Health Medical Center | 2020/1/28 |
|  |  |  |  |
| 4 | Comparison of efficacy and safety of three antiviral regimens in patients with mild to moderate novel coronavirus pneumonia (COVID-19): a randomized controlled trial | Chongqing Public Health Medical Center | 2020/1/28 |
|  |  |  |  |
| 5 | Clinical Controlled Trial for Traditional Chinese Medicine in the treatment of Novel Coronavirus Pneumonia (COVID-19) | China Academy of Chinese Medical Sciences | 2020/1/29 |
|  |  |  |  |
| 6 | Chinese Herbal medicine for Severe novel coronavirus pneumonia (COVID-19): a Randomized Controlled Trial | Dongzhimen Hospital Affiliated to Beijing University of Chinese Medicine | 2020/1/30 |

Source: Chinese Clinical Trial Registry (<http://www.chictr.org.cn>) and International clinical trials registry platform (<https://apps.who.int/trialsearch/default.aspx>)
